# A survey of depression and anxiety disorders in urban and rural Suriname

**DOI:** 10.1186/s12889-021-12454-5

**Published:** 2022-01-08

**Authors:** Raj Jadnanansing, Edwin de Beurs, Kajal Etwaroo, Matthijs Blankers, Rudi Dwarkasing, Jaap Peen, Vincent Lumsden, Robbert Bipat, Jack Dekker

**Affiliations:** 1 Center for Psychiatry in Suriname and Faculty of Social Science, Anton de Kom University of Suriname, Letitia Vriesdelaan 1 – 3, Paramaribo, Suriname; 2 Research Department, Arkin Mental Health Institute, Amsterdam, The Netherlands; 3 Department of Clinical Psychology, Faculty of Social Sciences, Leiden University, Leiden, The Netherlands; 4 Center for Psychiatry in Suriname and Department of Psychiatry, Anton de Kom University of Suriname, Paramaribo, Suriname; 5 Department of Physiology, Faculty of Medicine, Anton de Kom University of Suriname, Paramaribo, Suriname; 6 Department of Clinical Psychology, VU University Amsterdam, Amsterdam, The Netherlands

**Keywords:** Depression, Anxiety, Treatment gap, Urban, Rural

## Abstract

**Background:**

Suriname is a Low-middle income country consisting of diverse population groups. Epidemiological studies concerning mental disorders like depression and anxiety had not been conducted until 2015. The treatment gap for mental disorders in Low and middle-income countries (LMICs) may reach 76-80% as treatment is not always readily available. In this study, we estimate and compare the prevalence of potential cases of depression and anxiety, as well as the size of the treatment gap in a rural (Nickerie) and urban (Paramaribo) region of Suriname, a lower middle-income country.

**Methods:**

Subjects were selected by a specific sampling method of the national census bureau. The Center for Epidemiological Studies-Depression (CES-D) was used to assess depression. Generalized anxiety disorder was assessed with the Generalized Anxiety Disorder 7 (GAD-7) and The Agoraphobic Cognitions Questionnaire (ACQ), the Body Sensations Questionnaire (BSQ) were used to assess Panic disorder. The treatment gap was calculated by estimating the percentage of subjects with depression or anxiety that did not seek out professional help.

**Results:**

About 18% of the respondents from Nickerie and 16% from Paramaribo were at risk of depression and the established cut-off values of the instruments used indicate that about 3-4% in both regions may suffer from Generalized Anxiety Disorder. Women in both samples were most at risk of high anxiety about body sensations and maladaptive thoughts about panic. The treatment gap varies between 78 and 100% for the two disorders.

**Conclusions:**

A high depression rate has been found in both areas, especially among young females. In addition, a high treatment gap is noted which insinuates that more therapeutic strategies are required to tackle depression and anxiety in Suriname.

## Introduction

Depression is a common psychiatric disorder that has a negative effect on how an individual feels, thinks and behaves (The American Psychiatric Association, 2013). The Diagnostic and Statistical Manual of Mental Disorders (DSM-5) [1] indicates that the core symptoms of depression are a gloomy mood and a loss of interest and pleasure. The disorder usually presents as a lack of energy, disturbed sleep or appetite, low self-esteem [2] and aversion to activity, or apathy, which may affect a person’s thoughts, feelings, behaviour and sense of well-being [3]. Biological, environmental, and personal vulnerabilities interact to contribute to the development of depression, [4] which – when chronic – substantially impairs an individual’s ability to fulfil of day-to-day responsibilities, possibly even resulting in suicide [2]. The 12-month prevalence of major depression (DSM-IV) is thought to be between 5 and 6% worldwide [5].

About 85% of patients with depression also experience significant anxiety, while comorbid depression occurs in up to 90% of patients with anxiety disorders [6, 7]. Anxiety becomes problematic when it affects normal daily functioning. According to the DSM-5 [1], the condition shares features of excessive fear and anxiety, and related behavioural disturbances. An anxiety disorder is defined by excessive worry, hyperarousal and fear that is counterproductive and debilitating [8]. Both depression and anxiety are commonly linked to suicide [9, 10].

Anxiety and depressive disorders and symptoms significantly impair functioning in a number of areas including work, social life and health [11, 12]. In fact, the quality of life among depressed adults is more impaired than that of adults with diabetes, hypertension and chronic lung disease [13]. Similarly, anxiety has also been associated with a number of negative outcomes, including decreased work productivity, impaired work, family and social functioning, physical disability and even mortality [14–16].

On average, countries spend 2 1.7% of their health budgets on mental health, even though 14% of years lived with disability globally are known to be caused by depression and anxiety [17]. Depression is the most common mental illness, which affects more than 300 million people globally. Depression significantly contributes to the overall global burden of disease and is the single largest contributor to global disability [18, 19].

Depression and anxiety rates are generally higher in urban areas than in rural areas but there may be country-specific variations [17]. More than 50% of the global population lives in cities. This rise in urbanisation means that more people are exposed to risk factors associated with the social or physical environment in urban settings. This contributes to increasing stress, which is in turn negatively associated with mental health problems [20]. A systematic review [21] found that the annual prevalence of any given anxiety disorder varied between 2.4 and 29.8% across 44 countries (87 studies), and that the prevalence was higher in urban areas than in rural areas. Depending on severity, these disorders are managed with low-dose anti-depressants like serotonin reuptake inhibitors or anxiolytics like benzodiazepines [22]. However, psychological treatment and support is preferred for efficacy and price reasons [23, 24] and eHealth interventions using this approach have produced small but positive effects on symptom reduction [25]. Psychological treatment is not readily available in lower middle income countries (LMICs) and the treatment gap for mental disorders may consequently reach 76-80% in these countries [26]. Depression and anxiety make a significant contribution to the burden of disease and disability in low and middle income countries [27], thus an epidemiological study of depression and anxiety in a Low and Middle Income country is of great importance. In addition recent data on contributing social determinants for the risk of these disorders is scant and only a few recent studies are available. A systematic review of studies carried out in the Caribbean concluded in 2017 that the risk for depression follows the global trend [28]. A study among the diaspora in the USA found that they showed a lower risk of developing anxiety, but that on the other hand the disorder was more severe when developed [29]. Hence it is important to continuously determine the risk of developing these disorders and assessing their treatment gap.

The current study focuses on the prevalence of possible cases of depression and anxiety in a rural and an urban area in Suriname, a lower middle income country in South America. In this study we screen and identify people with a high risk of depression and anxiety, by screening the symptoms with the use of validated screening instruments. In addition to the indigenous people, the population consists of groups with different origins that can be traced back to Suriname’s plantation history: Maroons (21.7%), Creoles (15.7%), West Indians (27.4%), Javanese (13.7%), mixed race people (13.4%), people with another ethnicity (7.6%) and a small group of unknown ethnicities (0.6%) [30]. The capital of Suriname is the urban and generally industrial district, Paramaribo. Its rural counterpart is Nickerie, which is a mostly agricultural area. Several studies have found that the suicide rate in Suriname is higher than average but a possible relation with depression and/or anxiety has yet to be established [22]. No epidemiological studies of the depression and anxiety rates in the Surinamese population had been conducted prior to our study in 2015.

On the basis of major population surveys around the world, we expected higher depression and anxiety rates in the urban setting of Paramaribo than in the rural region of Nickerie. Gender differences for depression are not as apparent in rural contexts and they may even be absent in some societies [23, 24]. We will therefore compare gender differences in both the urban and rural contexts covered by this study.

The present study compared the prevalences of depression and anxiety in a rural and an urban area in Suriname and the associated factors. Nickerie and Paramaribo were selected for this study because of the urban and rural characteristics of the districts. Paramaribo is the only really urban area of Suriname and the distance between Paramaribo and Nickerie ensures that there is little to no urban influence on the latter. In addition, the treatment gap (respondents at risk but not receiving treatment) was also assessed in both areas.

## Methods

### Study design

In 2015 and 2016, we performed a large-scale survey of mental disorders and alcohol use disorder in the populations of Nickerie and Paramaribo, the first of its kind to be conducted in this country. The Centre for Psychiatry in Suriname (PCS) in Paramaribo initiated this large-scale survey in 2014 in collaboration with Arkin (a Dutch mental health service in Amsterdam) and the VU University in Amsterdam.

The collaboration resulted in a twinning project between the Surinamese PCS and Arkin, which funded the study [25]. For the purposes of the survey two districts were selected from the total of ten in the country: Paramaribo, Wanica, Nickerie, Coronie, Saramacca, Commewijne, Para, Marowijne, Brokopondo and Sipaliwini.

The results relating to alcohol and other substance use have been described in a separate paper [26]. The present paper looks at the results for depression and anxiety.

Suriname, a former Dutch colony, gained its independence and became a republic in 1975. In addition to the official language (Dutch), almost all inhabitants speak another language depending on their ancestral origins.

The country currently has a population of approximately 600,000 [30]. In addition, almost 400,000 people from at least three generations live in diaspora in the Netherlands. The result is intensive bilateral travel between these two countries.

### Participants/respondents

The respondents were recruited in the two areas using a specific sampling method using a large sample of 10% of all households in each participating resort, which are smaller population units in each district. The sampling method has been developed by the *Algemeen Bureau voor de Statistiek* of Suriname (ABS) (General Bureau of Statistics), where researchers and others can obtain adequate statistics for large scale research. This is the most common way for researchers in Suriname to obtain a representative population for their research. The ABS selected the addresses of the respondents aged 16-65 so as to ensure a balanced geographical distribution. The addresses where people no longer lived or that were abandoned were skipped and the interviewers then continued to the addresses of the houses to the right. This scenario had been taken into account before the field research began and, to ensure enough households were recruited, ABS had also supplied additional addresses. The final samples consisted of 1837 households for Paramaribo and 1026 for Nickerie. This ratio reflects the relative number of households in the two areas.

In the 23rd and the 42nd week of 2016, interviewers approached approximately 1100 and 2000 participants in Nickerie and Paramaribo, respectively. The respondents were required to give both written and oral consent for the study because not all of them were literate. The respondents who ultimately agreed to participate in the study were asked to complete a confidentiality form in order to safeguard their privacy. The interview was conducted in situ in a quiet location where the interviewer explained the aim of the study and asked the questions in the respondent’s preferred language. The respondents were given enough time to complete the questionnaire and all interviews were collected and submitted electronically. Government authorities and the district police made protection arrangements for the interviewers.

,After the selection, the respondents were required to give both written and verbal consent for the study because not all of them were literate. The respondents who ultimately agreed to participate in the study were asked to complete a confidentiality form in order to protect their privacy during this study. Interviews were conducted in a quiet place where the interviewer explained the aim of the study and the questions in the language referred by the respondents. Each respondent was given enough time to complete the questionnaire and all interviews were collected and submitted electronically. The interviews that could not be conducted and submitted electronically immediately, mostly due to network access issues, were conducted in writing and then transcribed and submitted electronically by the interviewer.

During this pilot study, the district commissioners (DCs), board supervisors and the police force provided assistance with the practicalities of collecting data. The DCs were informed about the purpose and design of the study beforehand, the police force organised physical protection for the interviewers and the board supervisors accompanied the interviewers as they knew the places and respondents well.

### Selection and training of interviewers

Before main data collection began in Nickerie and Paramaribo, a small pilot study with thirty respondents was completed in the regional health centre to validate the research tool. After each day of data collection in this pilot study, the group evaluated the difficulties that had been encountered and questionnaires with invalid or missing information were put aside immediately. The pilot study showed that it was necessary to translate the questionnaire into Hindustani, English and Surinamese. Although Dutch is the main language in both Nickerie and Paramaribo, the multi-cultural society of Suriname means that the inhabitants speak different languages and so the questionnaires sometimes had to be translated to make completion easier for respondents who spoke another language.

Another conclusion to emerge from the pilot study was that the students working as interviewers needed professional training. The students were interviewed individually by a psychiatrist, a researcher and a psychologist before being selected for training. Students with an academic background in psychology, with experience in similar interview work and students who spoke more than one language were preferred.

The training began after the completion of this selection procedure. It was delivered by experienced psychologists and psychiatrists and consisted of different components. Firstly, all the important terms such as depression, panic disorder and anxiety were discussed. Secondly, the students were given instructions about how to conduct and score the questionnaire.

In the last few days of the training, different dialects were practised with the students to prepare them for all types of situations in the field. The training lasted two weeks.

### Study size

Nickerie has a population of 34,233 and the final sample in Nickerie was 1026. Paramaribo has a population of 240,924 and the final sample was 1837. With the population size of both Nickerie and Paramaribo, an alpha of 0.05, and an error margin of 3%, with the aid of an online calculator (https://www.stat.ubc.ca/~rolling/stats/size/n2.html) it was determined that the sample size in Nickerie should be 1035 and in Paramaribo 1065. We oversampled to compensate for missing data. All the selected respondents were interviewed. If they were not available initially, the interviewer returned at another time to ensure that the questionnaire was completed.

#### Assessment/Instruments

##### Depression: the CESD

The Center for Epidemiological Studies Depression (CES-D) was designed to measure the level of depressive symptomatology in the general population [27]. Twenty items enquire about the frequency of symptoms in the past week, with response options ranging from 0 “Not at all” to 3 “Nearly every day”. The total sum score ranges from 0 to 60 and the cut-off point typically recommended for depression cases is 16 [31].

Sensitivity for major depression varies between 60 and 99% and specificity is between 73 and 94% for this cut-off point [32, 33], which we used in this study.

##### Anxiety

Two aspects of common anxiety were measured: generalized anxiety and excessive worry, and panic disorder. Generalized anxiety and worry was measured with the GAD-7 [34].

The GAD consists of seven items describing feelings such as “Trouble relaxing”, “Feeling nervous, anxious or on edge” and “Feeling afraid that something awful might happen”. Items are scored on a 4-point Likert scale (0 = not at all to 3 = nearly every day), resulting in a theoretical range in scores of 0 to 21. The GAD-7 has good reliability and good criteria, construct, factorial and procedural validity [34]. The cut-off point to establish a GAD is 16 or higher with optimal sensitivity (89%) and specificity (82%) [34]. As stated above, this cut-off was also used in this study.

Fear of fear was measured with the Agoraphobic Cognitions Questionnaire (ACQ) [35] and the Body Sensations Questionnaire (BSQ) [35]. The ACQ was devised to measure maladaptive thoughts about the possible consequences of panic (the cognitive aspect). Respondents rate the frequency of these thoughts when feeling anxious or frightened in fourteen items. Each item is rated on a 5-point Likert scale ranging from 1 (thought never occurs) to (thought always occurs). We used the total score (ACQ_TOT) in this study.

The scale discriminates well between patients and normal controls: Chambless et al. (1984) reported a mean score of 2.32 (SD = 0.66) for outpatients with agoraphobia, and 1.60 (SD = 0.46) for a community sample. The Dutch version of the ACQ was psychometrically evaluated by Arrindell (1993): it proved to be reliable (internal consistency Cronbach’s a > .82 for the ACQ) and test-retest reliability was good (Pearson PMC *r* > 0.79) [36]. A Dutch study [37] classified male respondents with a total score of more than 1.94 and female respondents with a total score of more than 1.86 [37] as highly anxious. We also use these cut-off points in this study.

The BSQ measures fear of the bodily sensations which are commonly experienced during anxiety and panic attacks. The BSQ consists of seventeen items, each of which describes a physical symptom such as dizziness, palpitations or breathlessness.

Items are rated on a five-point scale describing the level of anxiety they provoke ranging from 1 (not at all) to 5 (extreme). Chambless (1984) reported a mean score of 3.05 (SD = 0.86) for outpatients with agoraphobia and 1.80 (SD = 0.59) for a community sample.

The Dutch version of the BSQ was psychometrically evaluated by Arrindell (1993) and found to be reliable (internal consistency Cronbach’s a > .89 for BSQ) and test-retest reliability was good (Pearson PMC *r* > 0.79). For this study, we have used cut-off points from a Dutch study (Bouman, 1995) in which male respondents with a total score of more than 2.47 and female respondents with a total score of more than 2.40 were classified as being highly anxious about body sensations.

#### Treatment gap

After respondents completed the questionnaires on depression and anxiety, they were asked whether they had sought help from a General Practitioner or health professional for the reported psychological complaints. The treatment gap was calculated by determining the percentage of subjects with a depression or anxiety who did not seek help for related physical or mental disorders.

##### Statistics

Differences between socio-demographic characteristics in Paramaribo and Nickerie, and between respondents with or without risk of depression or anxiety, were compared using Chi-Squared (χ^2^) testing when confronted with categorical variables. All statistical analyses were conducted with SPSS (version 26; IBM; NY).

The quality assurance for the analyses was conducted with SPSS and Graphpad for Prism version 8.3. Data are presented as means (95% CI) unless noted otherwise.

## Results

A total of 1026 participants participated in the study in the Nickerie district. Of these, 593 were female and 433 were male. In Paramaribo, 1065 women and 772 men participated, a total of 1837 respondents. The entire sample for this study therefore consisted of 2863 participants. The response rate was almost 100% with the exception of a few missing respondents.

### Representativeness

Paramaribo has a population of 140,679: 51% female and 49% male, with 53% being younger than 40 years old. Nickerie has a population of 71,867: 47% female and 53% male, with 52% being younger than 40 years old.

Both samples included a significantly higher number of elderly women and a significant underrepresentation of younger men: 58% women (χ^2^ = 35.6;df = 1;*p* = 0.000) in Paramaribo and 59% in Nickerie (χ^2^ = 46.9; df = 1;*p* = 0.000), 54% of whom were older than 40 years (χ^2^ = 13.3;df = 1;*p* = 0.000).

### Demographics of the respondents

As stated above, the study sample consisted of 2863 people (*N* = 2863). In Nickerie, there were 593 female participants and 433 men, a total of 1026.

Most respondents from both Paramaribo (60.8%) and Nickerie (63.8%) had a low education level and worked full time (Paramaribo: 51.1% and Nickerie: 33%). The majority of the respondents in Paramaribo were single (47.7%) and the majority of the respondents from Nickerie were married (48.2%). Most respondents in Paramaribo (28.9%) consisted of Creole people and most respondents in Nickerie consisted of West Indians (61.9%).

Table 1 shows the risk for depression in both samples. The risk of depression in Nickerie is 18% and approximately 16% in Paramaribo. There are significant differences between the two samples in terms of gender, education level, marital status, ethnic background and daily activity. Risk factors include: female, low education, being widowed or divorced, and long-distance relationships. Furthermore, the Maroon subjects in the Paramaribo sample are most at risk. In the Nickerie sample, the West Indian, Javanese or different (those who chose ‘different’ as their ethnic background) subjects were most at risk.

**Table 1:** An overview of risk of depression based on demographic characteristics of both samples

Variable		Nickerie	***P***	Paramaribo	***P***
		*Possible cases of depression*		*Possible cases of depression*	
Age	<40	74 (15.7%)	0.141	160 (17.4%)	0.032*
	>40	115 (20.9%)		136 (14.8%)	
Gender	Male	86 (15.8%)	0.000*	89 (11.5%)	0.000*
	Female	121 (20.5%)		207 (19.4%)	
Education	Low	141 (21.7%)	0.001*	188 (20.1%)	0.002*
	Secondary	46 (12.9%)		58 (12.4%)	
	High	1 (7.7%)		18 (13.1%)	
Marital Status	Single	39 (14.3%)	0.000*	149 (17%)	0.000*
	Married	82 (16.8%)		54 (11.2%)	
	Widowed	15 (37.7%)		16 (25.8%)	
	Divorced	23 (39.7%)		19 (25.3%)	
	Concubinage	27 (18.4%)		46 (15.5%)	
	Long-distance relationship	3 (30%)		12 (27.9%)	
Ethnic background	West Indians	131 (20.7%)	0.000*	82 (19.3%)	0.045*
	Creole	14 (14.9%)		78 (14.7%)	
	Maroon	0 (0%)		53 (24.5%)	
	Javanese	24 (13.4%)		17 (19.1%)	
	Mixed	11 (14.1%)		54 (13.7%)	
	Other	9(31%)		12 (14.3%)	
Daily activity	Student/going to school	8 (8.5%)	0.000*	46 (19.7%)	0.033*
	Working part-time	15 (22.1%)		18 (17.3%)	
	Working full-time	56 (16.8%)		115 (12.2%)	
	Unemployed/jobseeker	25 (25%)		35 (31.5%)	
	Housewife/Houseman	71 (21.7%)		60 (21.9%)	
	Handyman	6 (13.6%)		8 (21.2%)	
	Retired	6 (14%)		14 (10.2%)	

The table also shows that age plays a role in Nickerie only: older respondents (>40 years) in Nickerie are most likely to suffer from depression.

Table 2 shows that the two samples have an equal risk of developing an anxiety disorder. Approximately 3.8% of the respondents in Nickerie and approximately 3.6% in Paramaribo may have GAD. There are significant differences between the two samples in terms of gender, education and daily activity. In terms of gender differences, we saw a higher anxiety risk for women in both Nickerie (5.4%) and Paramaribo (4.3%). In both samples people with low education have the most anxiety risk as opposed to the other education levels. Significant risk factors for anxiety disorders in both samples are: female, housewife, low education, divorced or widowed and unemployment. In addition, the West Indian respondents in Paramaribo are most at risk of an anxiety disorder. It should also be noted that those who are older than 40 are more likely to develop anxiety disorders in both Paramaribo (39%) and Nickerie (27%).

**Table 2:** An overview of the risk of anxiety (according to the GAD-7) based on demographic characteristics of both samples

Variable		Nickerie *Risk anxiety*	***P***	Paramaribo *Risk anxiety*	***P***
Age	<40	12 (2.6%)	0.127	27 (2.9%)	0.053*
	>40	27 (4.9%)		39 (4.3%)	
Gender	Male	7 (1.6%)	0.049*	20 (2.6%)	0.002*
	Female	32 (5.4%)		46 (4.3%)	
Education	Low	32 (4.9%)	0.012*	46 (4.9%)	0.026*
	Secondary	6 (1.7%)		10 (2.1%)	
	High	0 (0%)		2 (1.5%)	
Marital Status	Single	7 (2.6%)	0.056	24 (2.7%)	0.08
	Married	17 (3.4%)		19 (3.9%)	
	Widowed	4 (9.5%)		4 (6.5%)	
	Divorced	5 (8.6%)		7 (9.3%)	
	Concubinage	5 (3.4%)		10 (3.4%)	
	Long-distance relationship	1 (10%)		2 (4.7%)	
Ethnic background	West Indians	28 (4.4%)	0.037*	26 (6.1%)	0.527
	Creole	3 (3.2%)		13 (2.4%)	
	Maroons	0 (0%)		5 (2.3%)	
	Javanese	7 (3.9%)		5 (2.7%)	
	Mixed	0 (0%)		13 (3.3%)	
	Other	1 (3.4%)		4 (4.8%)	
Daily activity	Student/going to school	3 (3.2%)	0.000*	9 (3.8%)	0.019*
	Working part-time	0 (0%)		4 (3.8%)	
	Working full-time	9 (2.7%)		21 (2.2%)	
	Unemployed/jobseeker	5 (5%)		10 (9%)	
	Housewife/Houseman	22 (6.7%)		20 (7.3%)	
	Handyman	0 (0%)		1 (2.6%)	
	Retired	0 (0%)		66 (3.6%)	

Table 3 demonstrates that women in both samples are more at risk of high anxiety about body sensations (which was seen in approximately 19% of women and 10% of men). In the Nickerie sample, we found the highest risk among housewives (25%). Generally the results show that women and housewife/houseman have a higher odd of getting depressed. n the Paramaribo sample, students, the unemployed and housewives had a high risk (ranging from 17 to 21%). Furthermore, in the Paramaribo sample, the Maroons had the highest risk (24%). Young people under forty in Paramaribo also have a high risk (18%).

**Table 3:** Overview of the relationship between demographics and risk of high anxiety about body sensations

Variable		Nickerie		***P***	Paramaribo		***P***
		*Low*	*High*		*Low*	*High*	
Age	<40	395 (84%)	75(16%)	0.001*	760 (82.5%)	161 (17.5%)	0.943
	>40	463 (83.9%)	89 (16.1%)		808 (88.2%)	108 (11.8%)	
Gender	Male	387 (89.6%)	45 (10.4%)	0.000*	696 (90.2%)	76 (9.8%)	0.000*
	Female	471 (79.8%)	119 (20.2%)		872 (81.9%)	193 (18.1%)	
Education	Low	531 (81.7%)	119 (18.3%)	0.306	794 (84.8%)	142 (15.2%)	0.033*
	Secondary	315 (88%)	43 (12%)		397 (85.2%)	69 (14.8%)	
	High	11 (84.6%)	2 (15.4%)		123 (89.8%)	14 (10.2%)	
Marital Status	Single	238 (87.5%)	34 (12.5%)	0.051	725 (82.7%)	152 (17.3%)	0.322
	Married	403 (81.7%)	90 (18.3%)		431 (89%)	53 (11%)	
	Widowed	34 (81%)	8 (19%)		54 (87.1%)	8 (12.9%)	
	Divorced	47 (81%)	11(19%)		64 (85.3%)	11 (14.7%)	
	Concubinage	126 (86.3%)	20 (13.7%)		257 (86.8%)	39 (13.2%)	
	Long-distance relationship	9 (90%)	1 (10%)		37 (86%)	6 (14%)	
Ethnic background	West Indians	525 (83.2%)	106 (16.8%)	0.001*	368 (86.8%)	56 (13.2%)	0.277
	Creole	83 (87.4%)	12 (12.6%)		450 (84.7%)	81 (15.3%)	
	Maroon	6 (100%)	0 (0%)		165 (76.4%)	51 (23.6%)	
	Javanese	148 (81.8%)	33 (18.2%)		171 (91.4%)	16 (8.6%)	
	Mixed	66 (84.6%)	12 (15.4%)		344 (87.1%)	51 (12.9%)	
	Other	28 (96.6%)	1 (3.4%)		70 (83.3%)	14 (16.7%)	
Daily activity	Student/going to school	81 (86.2%)	13 (13.8%)	0.001*	189 (80.8%)	45 (19.2%)	0.000*
	Working part-time	59 (86.8%)	9 (13.2%)		86 (82.7%)	18 (17.3%)	
	Working full-time	293 (87.5%	42 (12.5%)		826 (88%)	113 (12%)	
	Unemployed/jobseeker	90 (89.1%)	11 (10.9%)		92 (82.9%)	19 (17.1%)	
	Housewife/Houseman	244 (75.3%)	80 (24/7%)		217 (79.2%)	57 (20.8%)	
	Handyman	42 (93.3%)	3 (6.7%)		33 (86.8%)	5 (13.2%)	
	Retired	38 (86.4%)	6 (13.6%)		125 (91.2%)	12 (8.8%)	

Table 4 shows that women in both samples were more at risk of maladaptive thoughts about panic (about 5 to 6%) than the men (about 2%). The highest risk in the Nickerie sample was seen in housewives (9%) and in the Paramaribo sample in handymen (18%)..

**Table 4:** Overview of the relationship between demographics and high risk of maladaptive thoughts about panic

Variable		Nickerie		***P***	Paramaribo		***P***
		*Low*	*High*		*Low*	*High*	
Age	<40	450 (96.2%)	18 (3.8%)	0.539	886 (96.2%)	35 (3.8%)	0.233
	>40	522 (94.6%)	30 (5.4%)		876 (95.6%)	40 (4.4%)	
Gender	Male	421 (97.9%)	9 (2.1%)	0.003*	753 (97.5%)	19 (2.5%)	0.001*
	Female	551 (93.4%)	39 (5.5%)		1009 (94.7%)	56 (5.3%)	
Education	Low	613 (96.9%)	36 (5.5%)	0.051	888 (94.9%)	48 (5.1%)	0.184
	Secondary	346 (96.9%)	11 (3.1%)		448 (96.1%)	18 (3.9%)	
	High	12 (92.3%)	1 (7.7%)		136 (99.3%)	1 (0.7%)	
Marital Status	Single	226 (97.8%)	6 (2.2%)	0.174	837 (95.4%)	40 (4.6%)	0.019*
	Married	468 (95.3%)	23 (4.7%)		473 (97.7%)	11 (2.3%)	
	Widowed	37 (88.1%)	5 (11.9%)		59 (95.2%)	3 (4.8%)	
	Divorced	51 (89.5%)	6 (10.5%)		69 (92%)	6 (8%)	
	Concubinage	140 (95.2%)	7 (4.8%)		283 (95.6%)	13 (4.4%)	
	Long-distance relationship	9 (90%)	1 (10%)		41 (95.3%)	2 (4.7%)	
Ethnic background	West Indians	592(94%)	38 (6%)	0.001*	404 (95.3%)	20 (4.7%)	0.205
	Creole	93 (97.9%)	2 (2.1%)		512 (96.4%)	19 (3.6%)	
	Maroon	6 (100%)	0 (0%)		199 (92.1%)	17 (7.9%)	
	Javanese	147 (97.2%)	5 (2.8%)		184 (98.4%)	3 (1.6%)	
	Mixed	76 (96.2%)	3 (3.8%)		380 (96.2%)	15 (3.8%)	
	Other	29 (100%)	0 (0%)		83 (98.8%)	1 (1.2%)	
Daily activity	Student/going to school	92 (97.9%)	2 (2.1%)	0.000*	222 (94.9%)	12 (5.1%)	0.004*
	Working part-time	68 (100%)	0 (0%)		96 (92.3%)	8 (7.7%)	
	Working full-time	319 (95.8%)	14 (4.2%)		920 (98%)	19 (2%)	
	Unemployed/jobseeker	98 (97%)	3 (3%)		102 (91.9%)	9 (8.1%)	
	Housewife/Houseman	296 (91.4%)	28 (8.6%)		257 (93.8%)	17 (6.2%)	
	Handyman	45 (100%)	0 (0%)		31 (81.6%)	7(18.4%)	
	Retired	43 (97.7%)	1 (2.3%)		134 (97.8%)	3 (2.2%)	

Table 5 presents the treatment gaps for the various disorders. They vary between 78 and 100%. The mean gap is about 90%. There are no significant differences between the two areas.

**Table 5:** Treatment gap for men and women in Paramaribo and Nickerie

	Nickerie				Paramaribo			
	Men (*N* = 429)		Women (*N* = 583)		Men (*N* = 772)		Women (*N*-1065)	
Risks for:	Total number with a risk	Not in treatment	Total number with a risk	Not in treatment	Total number with a risk	Not in treatment	Total number with a risk	Not in ttreatment
Major Depression risk (CES-D)	68	86.8%	119	93.3%	89	91%	207	89.9%
General Anxiety Risk (GAD-7)	7	100%	31	90.3%	20	85%	46	78.3%
High Maladaptive thoughts (ACQ)	9	88.9%	38	92.1%	19	78.9%	56	80.4%
High anxiety about body sensations (BSQ)	44	97.7%	118	93.2%	76	94.7%	193	94.8%

Table 6 shows that there was no difference between the two regions with regard to the risk of the disorders.

**Table 6:** Odd’s ratio with 95% confidence interval for the risks in Nickerie compared with Paramaribo

	Odd	Lower CI	Upper CI
Risks for:			
Major Depression risk (CES-D)	1.18	0.97	1.45
General Anxiety Risk (GAD-7)	1.06	0.71	1.59
High Maladaptive thoughts (ACQ)	1.11	0.90	1.38
High anxiety about body sensations (BSQ)	1.16	0.80	1.68

## Discussion

In summary, we found an 18% risk of depression in Nickerie and 16% in Paramaribo. The prevalence of the risk of general anxiety disorder was 3.8% in Nickerie and 3.6% in Paramaribo but the risk of women developing these disorders was higher in both regions. In addition, differences were found relating to civil status, ethnicity and education level. Moreover, women were more likely to develop somatic problems related to these disorders. We found a treatment gap of between 78 and 100% for the disorders studied.

In both areas, the risk of depression was significantly higher for women than men. We saw probable depression in about 20% of women and in 12% of men. In particular, women with a low level of education, widows, divorced and unemployed women had a higher risk. In low-income and developing countries, depression among women is associated with conditions that include limited economic and educational opportunities, economic difficulties, restricted autonomy, forced marriages, domestic violence, and low levels of family support [38].

The results of the current study indicate that probable depression is more prevalent in women (20%) than men (12%), the exact cause of this is unclear and worth further research. One study has found that gender differences relating to depression can be explained by the different social roles assigned by society to men and women [39]. Gender roles are defined in line with a society’s beliefs about differences between the sexes [40]. According to Yu [41], gender differences can be attributed to gender inequality to a significant extent. Psychosocial events such as coping style and the perceived stigma of mental illness all contribute to the increased vulnerability of women to depression as well [42]. These factors may have contributed to the higher percentage among the women of this study.

Turning to the stigma of mental illness: men tend to conform to traditional masculine norms, which are correlated with less help-seeking behaviour and more negative attitudes towards seeking psychological treatments [43]. Due to these traditional masculine norms, men who experience symptoms of depression may not actively seek help, the prevalence numbers among these men may hence go unreported. This could be the case in our study and could be an explanation for the higher depression and anxiety rates are Surinamese women than men.

The prevalence rates for anxiety (with the exception of high anxiety about body sensations) are much lower than the prevalence rates for depression in Nickerie and Paramaribo, but they are consistent with another study [44], a meta-analysis that found a life-time prevalence rate for GAF of 4.1% in women and 2.1% in men. We did find high percentages of respondents with high anxiety about body sensations. This is related to the high percentages of respondents with a likely depression.

The treatment gap of 90% on average is quite high but comparable with other LMICs. Improving treatment availability by establishing regional clinics could reduce this percentage to acceptable levels. We found that levels of possible depression are highest among West Indians.

In recent years, rural areas have reported higher suicide rates than urban areas worldwide and agricultural activity is a key characteristic of many rural areas [52]. Research shows that suicide rates in Nickerie are higher than those in Paramaribo (68 suicides from 2000-2003 in Nickerie) [32]. A possible explanation would be that depression (often preceding or associated with suicide) is more frequent in Nickerie than in Paramaribo. However, the current study shows that depression is as prevalent in Paramaribo as in Nickerie and further research may therefore seem beneficial.

## Conclusions

As the results indicate there is a risk of depression in both regions and that there is a treatment gap of 90%. Furthermore it has been concluded that probable depression is more prevalent in women (20%) than men (12%) and West-indians. Improving treatment availability by establishing regional clinics could remedy the high treatment gap.

## Data Availability

The data that support the findings of this study are available from the Center of Psychiatry in Suriname (PCS) but restrictions apply to the availability of these data, which were used under license for the current study and so are not publicly available. Data are, however, available from the authors upon reasonable request and subject to permission from the Center of Psychiatry in Suriname.

## Abbreviations

ABS: Algemeen Bureau voor de Statistiek (general Bureau of statistics)
ACQ: The Agoraphobic Cognitions Questionnaire
BSQ: The Body Sensations Questionnaire
CES-D: The Center for Epidemiological Studies-Depression
DC: District Commissioners
DSM-5: The Diagnostic and Statistical Manual of Mental Disorders
GAD-7: The Generalized Anxiety Disorder 7
LMICs: Low-middle-income countries
OR: Odds Ratio
PCS: The center for psychiatry in Suriname
WHO: World Health Organization

## References

[1] American Psychiatric Association (2013). Diagnostic and statistical manual of mental disorders (DSM-5®).

[2] Marcus M, Yasamy MT, Van Van Ommeren M, Chisholm D, Saxena S (2012). Depression: A global public health concern.

[3] Alqahtani A (2017). Prevention of Depression: A Review of Literature. J Depress Anxiety.

[4] England MJ, Sim LJ (2009). Depression in parents, parenting, and children: Opportunities to improve identification, treatment, and prevention. Depression in Parents, Parenting, and Children: Opportunities to Improve Identification, Treatment, and Prevention.

[5] Bromet E, Andrade LH, Hwang I, Sampson NA, Alonso J, De Girolamo G (2011). Cross-national epidemiology of DSM-IV major depressive episode. BMC Med.

[6] Gorman JM (1996). Comorbid depression and anxiety spectrum disorders. Depress Anxiety.

[7] Tiller JWG (2013). Depression and anxiety. Med J Aust.

[8] Simpson HB, Neria Y, Lewis-Fernández R, Schneier F (2010). Anxiety disorders: Theory, research and clinical perspectives.

[9] Takahashi Y (2001). Depression and suicide. Japan Med Assoc J.

[10] Kanwar A, Malik S, Prokop LJ, Sim LA, Feldstein D, Wang Z (2013). The association between anxiety disorders and suicidal behaviors: A systematic review and meta-analysis. Depress Anxiety.

[11] Wells KB, Stewart A, Hays RD, Burnam MA, Rogers W, Daniels M (1989). The Functioning and Well-being of Depressed Patients: Results From the Medical Outcomes Study. JAMA.

[12] Mendlowicz MV, Stein MB (2000). Quality of life in individuals with anxiety disorders. Am J Psychiatry.

[13] Wells KB, Sherbourne CD (1999). Functioning and Utility for Current Health of Patients With Depression or Chronic Medical Conditions in Managed, Primary Care Practices. Arch Gen Psychiatry.

[14] Brenes GA, Penninx BWJH, Judd PH, Rockwell E, Sewell DD, Wetherell JL (2008). Anxiety, depression and disability across the lifespan. Aging Ment Health.

[15] Kessler RC, Frank RG (1997). The impact of psychiatric disorders on work loss days. Psychol Med [Internet].

[16] Brenes GA, Kritchevsky SB, Mehta KM, Yaffe K, Simonsick EM, Ayonayon HN (2007). Scared to Death: Results From the Health, Aging, and Body Composition Study. Am J Geriatr Psychiatry [Internet].

[17] Purtle J, Nelson KL, Yang Y, Langellier B, Stankov I, Roux AVD (2019). Urban–rural differences in older adult depression: A systematic review and meta-analysis of comparative studies. Am J Prev Med.

[18] Vigo D, Thornicroft G, Atun R (2016). Estimating the true global burden of mental illness. Lancet Psychiatry.

[19] James SL, Abate D, Abate KH, Abay SM, Abbafati C, Abbasi N (2018). Global, regional, and national incidence, prevalence, and years lived with disability for 354 Diseases and Injuries for 195 countries and territories, 1990-2017: A systematic analysis for the Global Burden of Disease Study 2017. Lancet.

[20] Gruebner O, Rapp MA, Adli M, Kluge U, Galea S, Heinz A (2017). Risiko für psychische Erkrankungen in Städten.

[21] Baxter AJ, Scott KM, Vos T, Whiteford HA (2013). Global prevalence of anxiety disorders: a systematic review and meta-regression. Psychol Med.

[22] Graafsma T, Kerkhof A, Gibson D, Badloe R, Van de Beek LM (2006). High rates of suicide and attempted suicide using pesticides in Nickerie, Suriname, South America. Crisis.

[23] Astbury J (2001). Gender disparities in mental health.

[24] Tsai JL, Chentsova-Dutton Y, Gotlib IH, Hammen C (2002). Understanding depression across cultures. Handbook of Depression.

[25] Dwarkasing R, De Jonge M (2014). Onderzoek naar alcoholgebruik, angst en depressieve klachten in Suriname, en aanbieden van zorg op maat en geïndiceerde e-mental health.

[26] Jadnanansing R, Blankers M, Dwarkasing R, Etwaroo K, Lumsden V, Dekker J, et al. Prevalence of Substance Use Disorders in an Urban and a Rural Area in Suriname. Trop Med Health. 2021;49:12 10.1186/s41182-021-00301-7 PMC7852200 33526098

[27] Radloff LS (1977). The CES-D scale: A self-report depression scale for research in the general population. Appl Psychol Meas.

[28] Brown CR, Hambleton IR, Sobers-Grannum N, Hercules SM, Unwin N, Nigel Harris E (2017). Social determinants of depression and suicidal behaviour in the Caribbean: A systematic review. BMC Public Health.

[29] Himle JA, Baser RE, Taylor RJ, Campbell RD, Jackson JS (2009). Anxiety disorders among African Americans, blacks of Caribbean descent, and non-Hispanic whites in the United States. J Anxiety Disord [Internet].

[30] “Algemeen Bureau voor de Statistiek in Suriname.” Resultaten achtste (8e) volks – en woningtelling in suriname | Algemeen Bureau voor de Statistiek in Suriname [Internet]. [cited 2021 Jan 22]. Available from: https://statistics-suriname.org/nl/resultaten-achtste-8e-volks-en-woningtelling-in-suriname/

[31] Löwe B, Decker O, Müller S, Brähler E, Schellberg D, Herzog W (2008). Validation and standardization of the Generalized Anxiety Disorder Screener (GAD-7) in the general population. Med Care.

[32] Beekman ATF, Van Limbeek J, Deeg DJH, Wouters L, Van Tilburg W. Een screeningsinstrument voor depressie bij ouderen in de algemene bevolking: de bruikbaarheid van de Center for Epidemiologic Studies Depression Scale (CES-D). Tijdschr Gerontol Geriatr. 1994;25(3):95-103. 8036649

[33] Beekman ATF, Deeg DJH, Van Limbeek J, Braam AW, De Vries MZ (1997). Van Tilburg W. Brief communication.: criterion validity of the Center for Epidemiologic Studies Depression scale (CES-D): results from a community-based sample of older subjects in the Netherlands. Psychol Med.

[34] Spitzer RL, Kroenke K, Williams JBW, Löwe B (2006). A brief measure for assessing generalized anxiety disorder: the GAD-7. Arch Intern Med.

[35] Chambless DL, Caputo GC, Bright P, Gallagher R (1984). Assessment of fear of fear in agoraphobics: the body sensations questionnaire and the agoraphobic cognitions questionnaire. J Consult Clin Psychol.

[36] Arrindell WA (1993). The fear of fear concept: Stability, retest artefact and predictive power. Behav Res Ther.

[37] Bouman TK (1995). Kort instrumenteel: De Agoraphobic Cognition Questionnaire (ACQ). Gedragstherapie..

[38] Bhattacharya A, Camacho D, Kimberly LL, Lukens EP (2019). Women’s experiences and perceptions of depression in India: A metaethnography. Qual Health Res.

[39] González G, Vives A (2019). Work status, financial stress, family problems, and gender differences in the prevalence of depression in Chile. Ann Work Expo Heal.

[40] Blackstone AM, Miller JR, Lerner RM, Schiamberg Santa Barbara LB (2003). Gender Roles and Society.

[41] Yu S (2018). Uncovering the hidden impacts of inequality on mental health: a global study. Transl Psychiatry.

[42] Kulesza M, Raguram R, Rao D (2014). Perceived mental health related stigma, gender, and depressive symptom severity in a psychiatric facility in South India. Asian J Psychiatr.

[43] Levant RF, Wimer DJ, Williams CM (2011). An evaluation of the Health Behavior Inventory-20 (HBI-20) and its relationships to masculinity and attitudes towards seeking psychological help among college men. Psychol Men Masculinity.

[44] McLean CP, Asnaani A, Litz BT, Hofmann SG (2011). Gender differences in anxiety disorders: prevalence, course of illness, comorbidity and burden of illness. J Psychiatr Res.

